# A high density of ancient spliceosomal introns in oxymonad excavates

**DOI:** 10.1186/1471-2148-6-34

**Published:** 2006-04-25

**Authors:** Claudio H Slamovits, Patrick J Keeling

**Affiliations:** 1 Canadian Institute for Advanced Research, Botany Department, University of British Columbia, 3529-6270 University Boulevard, Vancouver, BC, V6T 1Z4, Canada

## Abstract

**Background:**

Certain eukaryotic genomes, such as those of the amitochondriate parasites *Giardia *and *Trichomonas*, have very low intron densities, so low that canonical spliceosomal introns have only recently been discovered through genome sequencing. These organisms were formerly thought to be ancient eukaryotes that diverged before introns originated, or at least became common. Now however, they are thought to be members of a supergroup known as excavates, whose members generally appear to have low densities of canonical introns. Here we have used environmental expressed sequence tag (EST) sequencing to identify 17 genes from the uncultivable oxymonad *Streblomastix strix*, to survey intron densities in this most poorly studied excavate group.

**Results:**

We find that *Streblomastix *genes contain an unexpectedly high intron density of about 1.1 introns per gene. Moreover, over 50% of these are at positions shared between a broad spectrum of eukaryotes, suggesting theyare very ancient introns, potentially present in the last common ancestor of eukaryotes.

**Conclusion:**

The *Streblomastix *data show that the genome of the ancestor of excavates likely contained many introns and the subsequent evolution of introns has proceeded very differently in different excavate lineages: in *Streblomastix *there has been much stasis while in *Trichomonas *and *Giardia *most introns have been lost.

## Background

One of the prominent features that distinguishes eukaryotic genomes from those of prokaryotes is the presence of spliceosomal introns. Introns are intervening sequences that are removed from expressed RNAs, in the case of spliceosomal introns through a series of transesterfications mediated by a large riboprotein complex called the spliceosome [1]. Spliceosomal introns are only known from eukaryotic nuclear genomes, and were the subject of intense controversy over their potential role in early gene origins and evolution, the so-called introns early versus late debate [2-4]. One of the interesting features of intron evolution that came to light during this debate was the large range in intron density. At one extreme, introns appeared to be lacking in several protist lineages that were, at the time, thought to be the earliest-branching eukaryotes. These lineages included diplomonads (e.g., *Giardia*) and parabasalia (e.g. *Trichomonas*).

The early-branching status of these organisms has since been undermined by a variety of data, and now diplomonads and parabasalia are thought to be part of a large assemblage of protists called excavates, which also includes trypanosomes, euglenids, and a number of parasitic and free living flagellate or amoeboflagellate lineages [5]. However, despite the accumulation of a considerable quantity of molecular data from both *Giardia *and *Trichomonas*, as well as the identification of proteins involving splicing in *Trichomonas *[6], evidence for introns in their genomes remained intriguingly elusive. Indeed, only recently were introns finally characterized in these organisms [7-9], and remain extremely rare. Only three introns have been found in *G. intestinalis *among thousands of known genes [8,9] and forty-one introns were identified in the *T. vaginalis *genome after exhaustive searches [7]. Information from excavates other than *Trichomonas *and *Giardia *is scarce, but overall there seems to be a generally low density of introns (with the possible exception of Jakobid flagellates based on one family of genes[10]). Moreover, other instances of non-canonical introns and splicing are known in excavates [11-13], as are systems where splicing machinery is put to a slightly different use such as trans-splicing [14-16].

One of the excavate groups about which we know very little are the oxymonads. Oxymonads are anaerobic flagellates found almost exclusively in association with animals, many in the guts of termites and wood-eating roaches [17]. This is the only group of amitochondriates for which secondary loss of mitochondria has not been yet demonstrated, but they are closely related to the flagellate *Trimastix*, which has a vestigial organelle, so a primary lack of mitochondria in oxymonads is unlikely. Mostoxymonads are not available in culture because they live in complex communities with other protists and prokaryotes. As a result, there are few molecular data available from any oxymonad, and no introns have been identified [18]. The oxymonad *Streblomastix strix *is asymbiont of the dampwood termite *Zootermopsis angusticollis *from North American Pacific coastal region. This species has a number of unusual morphological characters, including a peculiar long slender cell shape with deep longitudinal vanes which is apparently maintained by intimate association with epibiotic bacteria [19], So far, many copies of four genes (alpha-tubulin, beta-tubulin, HSP90, and elongation factor-1 alpha) have been characterized from *S. strix *[18], and the complete absence of introns from all sequences (a total of 19,888 bp) suggests the oxymonads might share low intron densities apparently common to excavates. Here, we have used the recent documentation of a rare non-canonical genetic code in *Streblomastix *[18] to identify 17 oxymonad genes from an environmental expressed sequence tag (EST) pool from the hindgut of *Zootermopsis*. The genomic DNA sequence for each mRNA was determined and we found that, in contrast to other amitochondriate protists and the limited data previously available for *Streblomastix*, a relatively high density of canonical spliceosomal introns. Moreover, a large proportion of these introns are shared in position with other distantly related eukaryotes, suggesting that they are ancient intron positions retained in oxymonads but lost in other excavates such as *Giardia *and *Trichomonas*.

## Results and discussion

### Identification of oxymonad sequences from ESTs

A total of 5,337 ESTs from a *Z. angusticollis *termite hindgut cDNAlibrary were sequenced and found to form 2,595 clusters of unique sequences. Overall, the sample was dominated by sequences of parabasalian origin (transcripts encoding parabasalian actin and actin-related proteins alone represented 32% of all ESTs). Moreover, there are few oxymonad sequences known outside this sample, so *Streblomastix *cDNAs could not be identified based on similarity to known genes (only 2 ESTs, corresponding to known *Streblomastix *alpha- and beta-tubulin sequences, were identified by BLASTX searches). Accordingly, we used the presence of a rare non-canonical genetic code in *Streblomastix *as a filter to identify at least those genes where non-canonical codons were sampled. In *Streblomastix*, TAA and TAG encode glutamine (Q) rather than stop as in the universal code [18], so all clusters were compared to public databases using BLASTX and examined individually for in frame stop codons, in particular at positions normally encoding glutamine. No other protist known to exist in *Z. angusticollis *has been shown to possess a non-canonical genetic code. The other prominent protists in this insect are parabasalia, which are not known to deviate from the universal genetic code and whose sequences are also easy to identify with BLASTX searches given their high similarity with *T. vaginalis *genomic sequences.

Using the non-canonical code as a filter, we were able to identify 17 protein-coding genes (Table 1), representing a major increase in the available sequence data from oxymonads. Formerly partial sequences of 4 protein coding genes were known from *Streblomastix*, and a handful of cDNAs were known from other species [18,20,21]. From this sample we recovered 8 complete protein-coding genes, an additional 5 genes missing only 1 to 30 codons at the N-terminus, and another three lacking from 100 to 160 codons at the N-terminus. In addition, a short fragment encoding 258 codons of the large protein UPF1 was severely truncated, but we failed to obtain more sequence. Complete or near-complete sequences included five ribosomal proteins (RPS7 and 9, RPL4, 18 and 21), alpha- and beta-tubulin, the nuclear transporter Ntf2, cyclophilin, a peptidyl-isomerase involved in assisting protein folding, and NAD-dependent glutamate dehydrogenase. Also, two versions of the cystein-protease Cathepsin B were obtained. Although related, these sequences exhibited several differences at the amino acid level, so they are likely to represent multiples copies of the gene. We also identified two copies of the carbon metabolism enzyme pyruvate phosphate dikinase (PPDK), the functional and evolutionary significance of which are discussed elsewhere [22]. One conserved hypothetical protein was also found to use the *Streblomastix *genetic code. This protein has homologues in diverse eukaryotes (e.g. *Arabidopsis thaliana *AAM67532), buthas no assigned function. UPF1 is a key member of nonsense-mediated decay (NMD). This protein may be of interest in *Streblomastix *because it is involved in a mechanism of mRNA surveillance devoted to eliminating defective transcripts, such as those carrying premature stop codons [23]. NMD has been described and studied in animals and yeasts, but not yet found in protists [24]. The presence of UPF1 in *Streblomastix *suggests NMD is used by oxymonads, and in organisms where stop codons are reassigned to encode amino acids. Finally, UAP56 is a member of the DEAD box family of RNA helicases that is associated with the spliceosome and intervenes in early steps of pre-mRNA splicing in mammals and yeasts, but is also linked to mRNA export [25,26]. Even in the absence of introns, the presence of UAP56 indicates the likely presence of the spliceosome in oxymonads, and therefore by extension introns as well.

**Table 1:** *S. strix *genes identified in this study. *Streblomastix *genes recovered from the *Z. angusticollis *hindgut RNA sample. For incomplete sequences, the number of missing amino acids were estimated from homologues from *Giardia *and/or *Trichomonas*. UPF1 shows extensive size variation among eukaryotic lineages (between 800 and 1600 amino acids, approximately), so it is difficult to determine how much sequence this fragment is lacking. ND: not determined.

**Protien name**	**Length(AA)**	**Introns**
RPS7	189	0
RPS9	188	3
RPL4	445	ND
RPL18	184	1
RPL21	146	2
Alpha-tubulin	450	0
Beta-tubulin	447	0
Cyclophillin	167	0
Cathespin B (1)	312	1
Cathespin B (2)	283	3
NAD-dependent glutamate dehydrogenase	446	1
Pyruvate phosphate dikinase (1)	779	1
Pyruvate phosphate dikinase (2)	784	0
UPF1	258	ND
UAP56/BAT1	272	2
Nuclear transport factor 2	123	2
Conserved hypothetical protein	203	5

### Introns in *Streblomastix *genes

Genomic DNA sequences were obtained for all *Streblomastix *coding regions identified from the cDNA library. Despite the fact that many alleles and loci representing four proteins were previously found to contain no introns, we found that most of the genes encoding these transcripts were interrupted by introns. In total, we found 21 introns in our sample of 17 genes with genes having as many as 5 introns (Table 1). Including previously known intronless EF-1 alpha and HSP90 genes (alpha and beta tubulin are included in our sample) [18], the overall density is 1.1 introns per gene. However this is likely to be an underestimation since some of our sequences are truncated and could contain further introns, and there is a bias favouring genes that are more often intronless (e.g. HSP90). This density is less than that observed in the relatively intron-rich mammals and plants, but comparable to many other eukaryotic genomes, and certainly much higher than *Giardia *and *Trichomonas *where only 3 and 41 introns have been detected despite very large quantities of genomic data [7,9].

Overall, the *Streblomastix *introns were found to exhibit characteristics typical of eukaryotic spliceosomal introns. Introns ranged from 46 to 229 bases (Table 2), but most were between 60 and 100 bases long, and the AT content was markedly higher than that of the coding sequence (Table 2). Spliceosomal introns are flanked by GT and AG dinucleotides in the vast majority of known introns, while about 0.1% are U12 AT-AC introns[27] and a very small proportion of known introns use other non-canonical splice boundaries. Interestingly, however, the first of only three introns from *G. intestinalis *to be discovered has CU-AG boundaries [8]. Of the twenty-one introns from *Streblomastix*, 20 featured canonical GT-AG boundaries, but one intron in *rps9 *was flanked by AC-AG splice sites. However, the *Streblomastix *intron is located very close to the start of the transcript, so we cannot exclude the possibility that this intron sequence is incomplete and a canonical boundary lies upstream.

**Table 2:** Basic features of the *S. strix *introns. Characteristics of 21 introns found in 17 *Streblomastix *genes analysed. Size and base composition are shown. GC% mRNA shows base composition of the coding sequence (excluding introns).

**Protien name**	**Intron**	**Size(bp)**	**%GC(Intron)**	**%GC(coding)**
RPS9	1	57	0.29	0.46
	2	88	0.38	
	3	57	0.3	
RPL18	1	60	0.25	0.49
RPL21	1	46	0.11	0.37
	2	66	0.09	
Cathespin B (1)	1	122	0.16	0.35
Cathespin B (2)	1	63	0.22	0.47
	2	66	0.18	
	3	91	0.15	
Glutamate Dehydrogenase	1	100	0.36	0.38
Pyruvate phosphate dikinase (1)	1	229	0.21	0.44
UAP56/BAT1	1	105	0.22	0.37
	2	168	0.15	
Nuclear transport factor 2	1	56	0.27	0.41
	2	58	0.2	
Conserved hypothetical	1	64	0.27	0.44
	2	61	0.31	
	3	102	0.23	
	4	54	0.3	
	5	69	0.22	

**Average**		**85**	**0.23**	**0.42**

**Figure 1:**
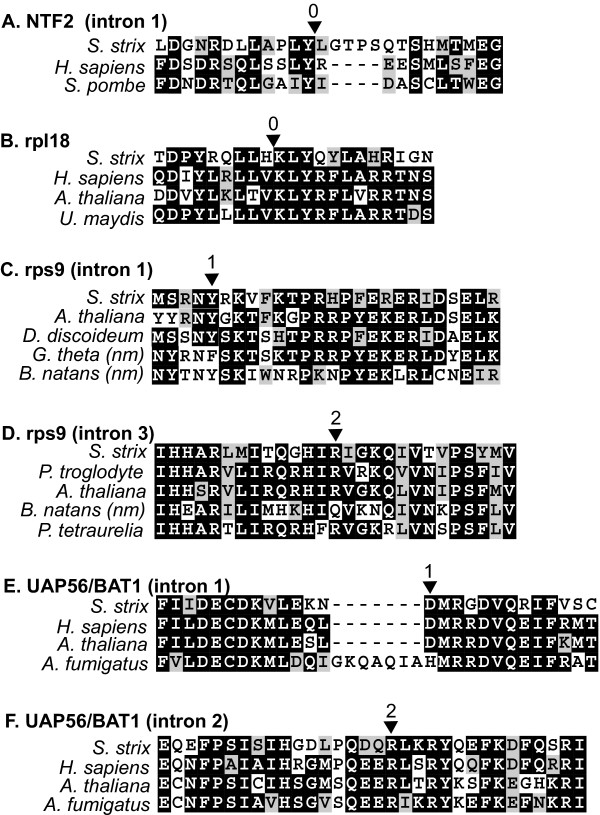
Examples of conserved intron positions between *Streblomastix* and other eukaryotes. In each case a section of the gene is shown aligned at the amino acid level, and the position of the intron found in all aligned sequences is indicated above by a triangle with a number indicating the phase (0, 1, or 2). Aligned sequences are from three unikont groups, animals (*H. sapiens* and *P. troglodytes*), fungi (*S. pombe*, *U. maydis* and *A. fumigatus*), and slime molds (*D. discoideum*), from one chromalveolate group, the ciliate (*P. tetraurelia*), and from three plantae groups, land plants (*A. thaliana*), green algae (*Bigelowiella natans* nucleomorph), and red algae (*Guillardia theta* nucleomorph).

We also inspected intron sequences to look for conserved features that may correspond to functional motifs. Although signals important for intron recognition and removal are not very well understood, some have been studied in certain detail, especially in mammals and yeasts. The branch-point is a sequence element required for lariat formation during splicing [28]. The mammalian branch point consensus sequence has been determined to be CURAY, where the A corresponds to the actual branching point. In yeast, the branch point sequence is more strictly defined as UACUAAC [29]. The plant branch point appears to be similar to that of mammals [30]. In all cases, the branch point is located near the 3' splice site, but the exact location varies. In contrast, the putative branch point found in the three introns of *Giardia *(ACURAC) is located directly adjacent to the 3' splice site [9]. Likewise, the potential branch points in *Trichomonas *are invariably ACUAAC and are also adjacent to the 3' splice site [7]. The apparently strict requirement for proximity between the branch point and 3' splice site is rare in metazoa and yeast, but common to *Trichomonas *and *Giardia*. This led to the suggestion that the branch point and 3' splice site recognition could be combined in these species [7]. Aligning the regions around the 5' splice site of all *Streblomastix *introns (Figure 1) reveals highly conserved A, U and G residues at positions +3 to +5, respectively. This is in good agreement with the first 5 positions of the yeast 5' splice site (typically GUAUGU), suggesting that interaction with U1 snRNA is conserved. At the 3' splice site no branch point motifs like those of *Giardia *or *Trichomonas *were observed, although the -1 position (adjacent to the AG dinucleotide) was invariably a pyrimidine and the region is T-rich. Overall, branch point specification in *Streblomastix *introns is probably different from that of *Giardia *or *Trichomonas*. Under the assumption that these lineages are related it is possible that the peculiar features observed in *Giardia *and *Trichomonas *may be a consequence of secondary implification in their spliceosomal apparatus.

### Conservation of intron positions in oxymonad genes

**Figure 2:**
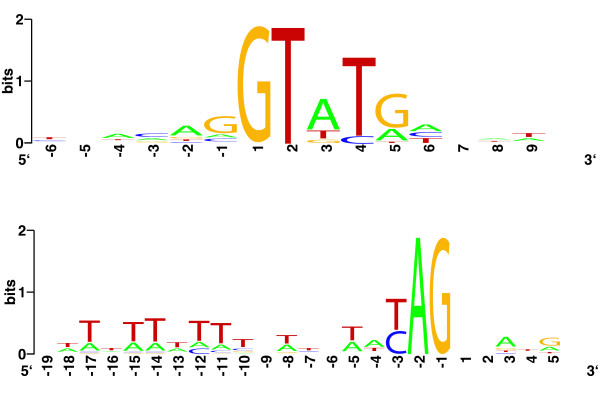
Sequence logos showing conservation at intron borders. Top: 5’ splice site (position 1) and surrounding sequence. Bottom: 3’ splice site (-1) and surrounding sequence. Logos were made using Weblogo ().

*Streblomastix *intron-containing genes were compared to homologues from other eukaryotes, and surprisingly more than half of the *Streblomastix *intron positions were shared with members of at least two different eukaryotic supergroups, unikonts and plants (where the best sampling of intron-containing genes exists), and in one case also with a chromalveolate (for six examples, see Figure 2). This suggests these are relatively ancient introns and perhaps date back to the last common ancestor of all eukaryotes. This degree of conservation is high, taking into account data such as those of Rogozin et al., who calculated that approximately 20% of the introns in *Plasmodium *are shared by at least one of the other genomes analysed (Human, *Anopheles*, *Drosophila*, *Caenorhabditis, Arabidopsis*, *Schizosaccharomyces *and *Saccharomyces*), and that 25% of the human introns are shared by *Arabidopsis *[31]. It is also possible that shared intron positions are due to independent gains, but it is very unlikely that the observed level of shared positions (about 50%) resulted from parallel gains, in particular in the many cases where the intron is found in several of the major lineages of eukaryotes. Whether intron gains or losses predominate in eukaryotic evolution is still a subject of controversy. Recently, several studies using different analytic approaches and datasets addressed this question with varied results, but in all cases, they show that ancestral conservation accounts for the large majority of shared positions [32-36]. The degree of conservation observed in *Streblomastix *intron positions suggests two things. First, it suggests that the ancestor of excavates was relatively intron rich and retained a large number of ancient introns, many of which were subsequently lost in the genomes where we have the most information, such as trypanosomes, *Giardia *and *Trichomonas*. This assumes the relationship between oxymonads and other hypothesized excavates is correct, but this is not certain and oxymonads lack the morphological trait used to define excavates (the ventral groove). However, other ultrastructural characters [37] as well as molecular phylogenies have shown a close affiliation between oxymonads and *Trimastix *[38], a free living flagellate that does have excavate characteristics [5,39]. Multi-gene phylogenies also lend additional support for a common origin of the lineages leading to oxymonads, diplomonads and parabasalia [21]. The second implication of this data is that intron gain and loss have taken place very slowly in the lineage leading up to *Streblomastix*: if intron turnover were rapid, then we would expect a low proportion of ancient introns to remain unless ancient intron positions were under some selection to be retained. While this is probably true in a few individual cases where introns have acquired some function in the control of gene expression, there is presently no evidence either for or against this as a common feature of ancient introns. None of these shared introns are known from either *Giardia *or *Trichomonas*, so any potential function is clearly dispensable, although it is interesting to note that the *rps9 *intron 1 has been retained by the *G. theta *nucleomorph, which is very intron poor, having kept a total of only 17 introns [40].

## Conclusion

The present sampling of protein-coding gene sequences from *Streblomastix *suggests that oxymonad genomes contain a relatively large number of canonical splicesomal introns, many of which are at ancient conserved positions. This is in contrast to the better studied excavate genomes such as those of kinetoplastids, *Giardia *and *Trichomonas *where canonical spliceosomal introns are either rare or have been co-opted in specific ways, such as the spliced leaders in euglenozoa. The fact that many *Streblomastix *introns are ancient shows that the genome of the ancestor of these organisms, and indeed probably all extant eukaryotes, contained many introns and that the intron-poor state found in *Giardia *and *Trichomonas *is more likely independently derived.

## Methods

### cDNA library construction and EST sequencing

Termites were collected from a rotten log in Point Grey, Vancouver, Canada. The whole hindgut content of about 60 individuals of *Zootermopsis angusticollis *from a single colony was collected and total RNA was extracted using TRIZOL (Invitrogen). A directionally cloned cDNA library was constructed (Amplicon Express) and 5,337 clones were sequenced from the 5' end. ESTs were trimmed for vector and quality, and assembled into clusters by PEPdb .

### Identification and genomic characterisation of *Streblomastix *genes

*Streblomastix *sequences were recovered from EST data by identifying protein coding sequences containing in-frame TAA and TAG stop codons. Putatively stop-coding containing mRNAs were re-sequenced in both strands. In cases where cDNA clones were truncated, the sequences were extended by means of 3' and 5' RACE (Ambion) using total termite hindgut RNA. The genomic sequence for each mRNA was amplified using specific primers corresponding to the ends of each complete or partial cDNA and PCR-amplified using genomic DNA purified from the termite hindgut content. All PCR products were cloned using TOPO and sequenced both strands. Accession numbers for new sequences are [genbankDQ363664, genbankDQ363665, genbankDQ363666, genbankDQ363667, genbankDQ363668, genbankDQ363669, genbankDQ363670, genbankDQ363671, genbankDQ363672, genbankDQ363673, genbankDQ363674, genbankDQ363675, genbankDQ363676, genbankDQ363677, genbankDQ363678, genbankDQ363679].

## Authors' contributions

CHS analysed the EST data, performed PCR and sequencing, and examined conservation of intron positions in other organisms. PJK collected the termites and purified RNA for library construction. Both authors participated in the writing and editing of the manuscript. All authors read and approved the final manuscript.
